# Early-Onset Type 2 Diabetes as a Risk Factor for End-Stage Renal Disease in Patients With Diabetic Kidney Disease

**DOI:** 10.5888/pcd17.200076

**Published:** 2020-07-02

**Authors:** Li Zheng, Xiangjun Chen, Ting Luo, Xi Ran, Jinbo Hu, Qingfeng Cheng, Shumin Yang, Jinshan Wu, Qifu Li, Zhihong Wang

**Affiliations:** 1 Department of Endocrinology, The First Affiliated Hospital of Chongqing Medical University, Chongqing, China

## Abstract

**Introduction:**

Compared with the typical onset of type 2 diabetes in middle age or older, type 2 diabetes with early age of onset has a higher risk of diabetes-related complications. It is unclear whether the early age of diabetes diagnosis would affect the development of end-stage renal disease (ESRD) in patients with diabetic kidney disease (DKD) who are at higher risk of ESRD.

**Methods:**

We enrolled 1,111 type 2 diabetes patients with DKD in this study. We used the age at diabetes diagnosis of younger than 40 years to define early-onset diabetes and 40 years or older to define late-onset diabetes. Medical history, anthropometry, and laboratory indicators were documented. ESRD was defined by estimated glomerular filtration rate (eGFR) of less than 15 mL/min/1.73 m^2^ or dialysis. Logistic regression analysis was used to explore the association between early-onset diabetes and ESRD.

**Results:**

Early-onset diabetes patients had a longer diabetes duration, higher body mass index, and worse blood lipid metabolism profile. Compared with late-onset diabetes patients, patients with early-onset diabetes had a prevalence of ESRD that was twofold higher (9.2% vs 4.3%; *P* = .009). Univariate analysis showed that early-onset diabetes was a risk factor for ESRD in patients with DKD (*P* < .05). In multivariate logistic regression analysis, even after adjusting for sex, traditional metabolic factors, drug factors, and diabetes duration, the risk of ESRD in patients with early-onset diabetes was still 3.58-fold higher than in subjects with late-onset (95% CI, 1.47–8.74; *P* = .005).

**Conclusions:**

In patients with DKD, early-onset type 2 diabetes is an independent risk factor of ESRD.

**Summary**

**What is already known on this topic?**

Compared with the type 2 diabetes of typical onset in middle age or older, type 2 diabetes with early age of onset has a higher risk of diabetes-related complications.

**What is added by this report?**

In patients with diabetic kidney disease (DKD), the prevalence of end-stage renal disease (ERSD) in the early-onset group was twofold higher than it was in the late-onset group. Early-onset diabetes is an independent risk factor for ESRD in patients with DKD.

**What are the implications for public health practice?**

Prevention strategies for diabetic complications should emphasize closer attention to patients with DKD and early-onset diabetes. More stringent metabolic targets should be applied to delay DKD progression.

## Introduction

End-stage renal disease (ESRD) is a global health problem with increasing prevalence (1). Diabetic kidney disease (DKD) is the leading cause of ESRD, accounting for approximately 50% of cases in developed countries worldwide (2,3). The progress of DKD to ESRD negatively affects the treatment of diseases and increases the economic burden of individuals and societies (4,5). Therefore, early detection of risk factors for DKD progressing to ESRD is a promising strategy for reducing related mortality and social economic burden.

Duration of diabetes has been considered a strong factor in the development of vascular complications of type 2 diabetes (6). Some studies have found that a subset of patients with longer diabetes duration are characterized by an earlier age of diabetes onset. Compared with patients with type 2 diabetes of typical onset in middle age or older, patients with type 2 diabetes with early age of onset (early-onset diabetes) have poorer metabolic control; higher risks for chronic kidney disease, retinopathy, and neuropathy (7–9); higher incidence of nonfatal cardiovascular disease (10); and higher standardized mortality (11).

Whether early-onset diabetes will affect DKD progressing to ESRD is still unknown. One study showed that early-onset diabetes has higher rates of macroalbuminuria (12). The appearance of macroalbuminuria has been viewed as the beginning of progressive renal function loss and impending failure or ESRD. Therefore, early-onset diabetes may promote the occurrence of ESRD. A prospective study found that younger onset of diabetes (onset age <20 years) increased the risk of ESRD, even after adjusting for age and sex (7). However, that study focused on type 2 diabetes in adolescents. It is unknown whether this conclusion is consistent in adult-onset type 2 diabetes patients with DKD who are at higher risk of ESRD. Therefore, we investigated 1,111 type 2 diabetes patients with DKD to determine the relationship between early-onset diabetes and the risk of ESRD.

## Methods

### Study design and study population

This is a retrospective cross-sectional study from The First Affiliated Hospital of Chongqing Medical University in China. We enrolled 1,111 inpatients (686 men and 425 women) with diagnosed type 2 diabetes with DKD who were in the hospital from January 1, 2014, to June 25, 2018. They were screened from the electronic medical record system based on inclusion and exclusion criteria. The inclusion criteria were the following:

- type 2 diabetes meeting the 1999 World Health Organization's diagnostic criteria for type 2 diabetes (13): 1) fasting plasma glucose (FPG; fasting defined as no caloric intake for at least 8 h) of 126 mg/dL or higher (7.0 mmol/L or higher); or 2) 2-h plasma glucose value of 200 mg/dL or higher (11.1 mmol/L or higher) during a 75-g oral glucose tolerance test; or 3) a patient with classic symptoms of increased thirst, urine volume, unexplained weight loss, and a random plasma glucose of 200 mg/dL or higher (11.1 mmol/L or higher);
- aged 18 years or older;
- have at least 1 previous inpatient medical record for diabetes; and
- diagnosed with DKD, defined as meeting at least 1 of the following (14): 1) random urinary microalbumin creatinine ratio (UACR) was 30 mg/g or higher creatinine in at least 2 of 3 tests; or 2) estimated glomerular filtration rate (eGFR) was 60 mL/min/1.73m^2^ or less.

The exclusion criteria were 1) patients without information about diabetes duration; 2) patients with half or more missing data on all key variables; 3) pregnant or lactating women; 4) patients with type 1 diabetes or other special types of diabetes; 5) patients definitely diagnosed with other types of chronic renal diseases such as IgA nephropathy or membranous nephropathy; 6) patients with urinary tract obstruction, urinary tract injury, urinary tract infection, or other conditions affecting urinary albumin; 7) patients who need long-term glucocorticoid treatment of other chronic diseases; and 8) patients with immune dysfunction, severe infection, or malignant tumor history. Patients were defined as having early-onset diabetes and late-onset diabetes based on the age at diagnosis of diabetes of less than 40 years or 40 years or older, respectively (15,16). The selection of 40 years as a cutoff was based on the use of similar age strata (20–39 years, 40–59 years, and 60–79 years) in the latest International Diabetes Federation estimates of world diabetes prevalence (17). Previous studies have used 40 years as an age threshold for young-onset diabetes (15,18), especially the 2 large sample surveys in Mainland China and Hong Kong (10,12).

### Clinical procedures

We reviewed clinical information including age, sex, history of diabetes (including duration of diabetes and age at first diagnosis of diabetes), smoking and drinking history, the history of concomitant diseases or diabetes complications, and medication history. All patients underwent physical anthropometry measurements including weight, height, systolic blood pressure (SBP), diastolic blood pressure (DBP), and body mass index (BMI), which was calculated by dividing weight in kilograms by height in meters squared. Laboratory assessments, including plasma glucose levels, glycosylated hemoglobin (HbA_1c_), total cholesterol (TC), triglyceride (TG), high density lipoprotein cholesterol (HDL-C), low density lipoprotein cholesterol (LDL-C), serum creatinine (sCr), urinary creatinine, and albumin were done in our hospital. Details of the measurement method were given in our previous study (19). UACR was calculated by urinary albumin/creatinine ratio. eGFR was estimated by using the Modification of Diet in Renal Disease Study equation (men: eGFR = 186 × sCr^−1.154^ × year^−0.203^; women: eGFR = 186 × sCr^−1.154^ × year^−0.203 × 0.724^) (20). ESRD was defined as dialysis or by eGFR lower than 15 mL/min/1.73m^2^ (12).

### Statistical analysis

Statistical analysis was performed by using the Statistical Package for Social Science (SPSS) version 20.0 (IBM). Data were expressed as mean (standard deviation [SD]) when the sample distribution is approximately normal. If the data were not a normal distribution model after testing with a quantile-quantile map, we used median (interquartile range) for expression. Continuous variables between 2 groups were compared by using the *t* test or Mann–Whitney *U* test. Categorical variables were reported as frequencies (proportions), and χ^2^ tests were used for group comparisons. We conducted univariate logistic analysis to investigate risk factors for ESRD in patients with DKD and multivariate logistic regression analysis to determine whether early-onset diabetes could be an independent risk factor for ESRD. Four models were analyzed to adjust for confounding factors, and the included variables were significant indicators in the univariate logistic analysis and indicators suggested by previous studies. Model 1 was adjusted for sex. Model 2 was further adjusted for BMI, blood pressure, blood lipids, HbA_1c_, and history of smoking, drinking, and hypertension in addition to the variable in model 1. Model 3 was adjusted for drug factors on the basis of model 2. Model 4 further adjusted for duration of diabetes in addition to the variables in model 3. Bilateral *P* values less than .05 were considered significant.

## Results

### Clinical features of patients

The mean (SD) age of the 1,111 patients with DKD was 63.6 (10.8) years, with a mean (SD) diabetes duration of 11.9 (7.2) years. A total of 152 (13.7%) patients had early-onset type 2 diabetes. Compared with late-onset diabetes patients, early-onset diabetes patients were more likely to be male, younger, have had earlier onset, have had a longer diabetes duration, and have had a history of smoking and drinking. They had a higher BMI, had higher DBP, worse blood lipid profile (higher TC, TG, and LDL-C) and blood glucose metabolism (higher HbA_1c_ concentrations), and had higher UACR and eGFR. Patients with early onset diabetes were less likely to be treated with oral hypoglycemic drugs, antihypertensive drugs, and renin-angiotensin system (RAS) blockers. Although there were higher proportions of insulin therapy and lower proportions of lipid-lowering drugs in the early-onset diabetes group, these differences were not significant (Table 1).

**Table 1:** Clinical Characteristics of Patients With Early-Onset and Late-Onset Type 2 Diabetes, The First Affiliated Hospital of Chongqing Medical University, China, 2014–2018

Characteristic	Overall (N = 1,111)	Onset		
		Early (n = 152)	Late (n = 959)	*P* Value
Age, mean (SD), y	63.6 (10.8)	48.4 (8.4)	66 (9.1)	<.001
Age of diabetes onset, mean (SD), y	52.1 (11.0)	34.6 (3.8)	54.9 (9.1)	<.001
Duration of diabetes, mean (SD), y	11.9 (7.2)	13.6 (8.0)	11.6 (7.1)	.001
Male, n (%)	686 (61.7)	116 (76.3)	570 (59.4)	<.001
Hypertension, n (%)^a^	763 (69.2)	79 (53.0)	684 (71.8)	<.001
Smoking, n (%)	531 (47.8)	99 (65.1)	432 (45.0)	<.001
Drinking, n (%)	436 (39.2)	86 (56.6)	350 (36.5)	<.001
Body mass index^b^, mean (SD)	25.1 (3.6)	25.8 (3.8)	25.0 (3.6)	.013
Systolic blood pressure, mean (SD), mmHg	143.7 (22.5)	140.9 (24.6)	144.1 (22.1)	.010
Diastolic blood pressure, mean (SD), mmHg	79.6 (13.6)	86.6 (13.6)	78.4 (13.2)	<.001
Total cholesterol, mean (SD), mmol/L	4.2 (1.3)	4.5 (1.4)	4.1 (1.2)	<.001
Total triglyceride, mean (SD), mmol/L	2.1 (1.9)	2.7 (2.4)	2.0 (1.7)	<.001
High density lipoprotein cholesterol, mean (SD), mmol/L	1.1 (0.4)	1.0 (0.3)	1.1 (0.4)	.21
Low density lipoprotein cholesterol, mean (SD), mmol/L	2.5 (1.1)	2.7 (1.1)	2.4 (1.0)	.009
HbA_1c_, mean (SD), %	9.1 (2.4)	9.5 (2.4)	9.0 (2.4)	.04
Urinary microalbumin creatinine (Cr) ratio, median (interquartile range), mg/g Cr	194.1 (69.1–753.7)	221.2 (77.6–1,250.0)	184.1 (67.2–695.0)	.03
Estimated glomerular filtration rate, mean (SD)	67.9 (34.6)	80.3 (43.0)	66.0 (32.7)	<.001
Oral hypoglycemic drugs, n (%)	640 (57.6)	74 (48.7)	566 (59.0)	.02
Insulin therapy, n (%)^a^	691 (62.9)	101 (67.8)	590 (62.1)	.18
Antihypertensive drugs, n (%)^a^	672 (61.6)	72 (48.3)	600 (63.7)	<.001
RAS blockers, n (%)^a^	408 (37.4)	41 (27.5)	367 (39.0)	.007
Lipid-lowering drugs, n (%)	216 (19.4)	22 (14.5)	194 (20.2)	.11
End stage renal disease, n (%)	55 (5.0)	14 (9.2)	41 (4.3)	.009

Abbreviations: CI, confidence interval; HbA_1c_, glycated hemoglobin; RAS blockers, renin-angiotensin system blockers, including angiotensin converting enzyme inhibitors and angiotensin receptor antagonists.

a Data were the effective percentage; the number of patients with missing data has been subtracted from the denominator.

b Calculated as weight in kilograms divided by height in meters squared.

Patients with early-onset diabetes had higher prevalence of ESRD (9.2%) than patients with late-onset diabetes (4.3%) (*P* = .009). Furthermore, we observed that the mean (SD) age of diabetes onset was significantly younger in patients with ESRD than those without ESRD (47.5 [11.8] years vs 52.4 [11.0] years, *P* = .001) (Figure), and the percentage of early-onset diabetes in patients with ESRD was higher than it was in those without ESRD (25.5% vs 13.1%, *P* = .009, Pearson χ^2^ test).

**Figure:**
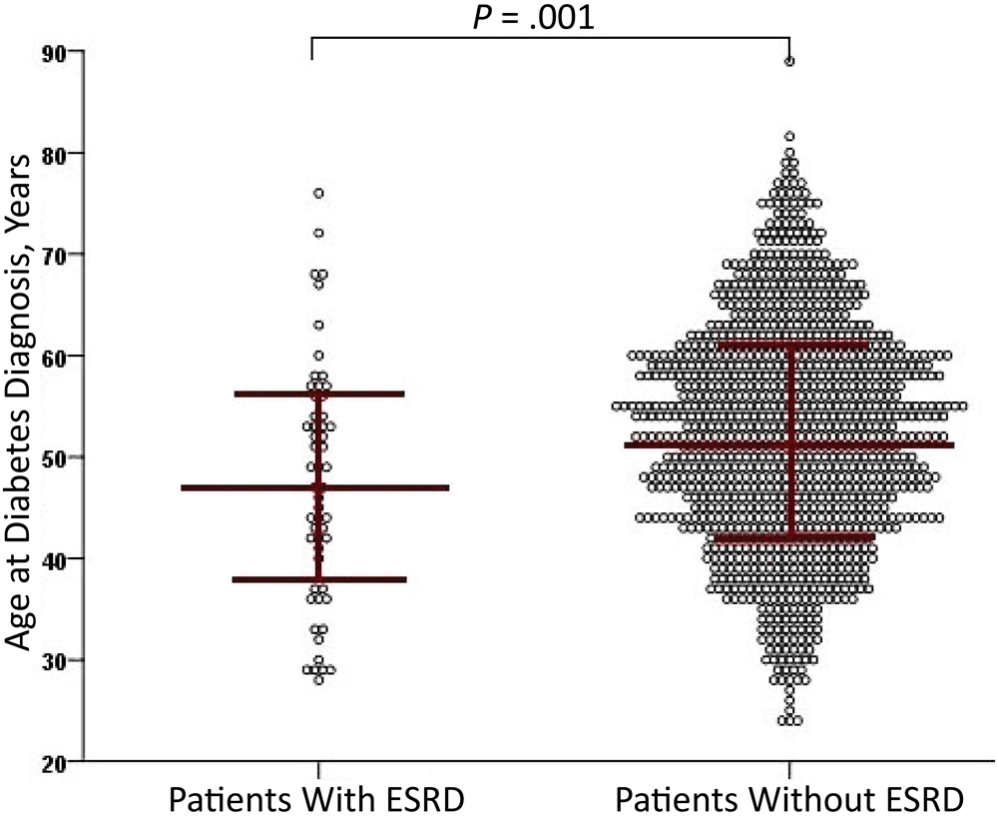
Age at diabetes diagnosis in 1,111 patients with or without end-stage renal disease (ESRD), The First Affiliated Hospital of Chongqing Medical University, China, 2014–2018. Circles represent patients; red lines represent mean and standard deviation.

### Risk factors for ESRD in patients with DKD

Univariate logistic analysis was used to identify the risk factors for ESRD in patients with DKD. Risk factors for ESRD were early-onset type 2 diabetes (odds ratio [OR], 2.27 [95% CI, 1.21–4.28]; *P* = .011), duration of diabetes (OR, 1.07 [95% CI, 1.03–1.11]; *P* < .001), history of hypertension (OR, 3.81 [95% CI, 1.62–8.98]; *P* = .002), SBP (OR, 1.02 [95% CI, 1.01–1.03]; *P* = .001), use of insulin therapy (OR, 3.16 [95% CI, 1.53–6.53]; *P* = .002), and use of antihypertensive drugs (OR, 3.78 [95% CI, 1.76–8.08]; *P* = .001). However, we found that HbA_1c_ concentrations, use of oral hypoglycemic drugs, and use of RAS blockers were protective factors for ESRD, and age, sex, blood lipids, BMI, and use of lipid-lowering drugs were not related to the development of ESRD (Table 2).

**Table 2:** Univariate Logistic Regression Analysis for End Stage Renal Disease in Patients With Diabetic Kidney Disease, The First Affiliated Hospital of Chongqing Medical University, China, 2014–2018

Variable	Odds Ratio (95% Confidence Interval)	*P* value
Age, y	1.00 (0.97–1.02)	.80
Sex, male vs female	1.00 (0.57–1.75)	.99
Onset, early vs late	2.27 (1.21–4.28)	.011
Duration of diabetes, y	1.07 (1.03–1.11)	<.001
History of hypertension	3.81 (1.62–8.98)	.002
HbA_1c_, %	0.72 (0.62–0.85)	<.001
Total cholesterol, mmol/L	1.09 (0.89–1.33)	.42
Total triglyceride, mmol/L	0.88 (0.72–1.08)	.22
Low density lipoprotein cholesterol, mmol/L	1.15 (0.91–1.45)	.26
Systolic blood pressure, mmHg	1.02 (1.01–1.03)	.001
Diastolic blood pressure, mmHg	0.99 (0.97–1.01)	.46
Body mass index^a^	1.01 (0.93–1.09)	.89
Use of oral hypoglycemic drugs	0.19 (0.10–0.36)	<.001
Use of insulin therapy	3.16 (1.53–6.53)	.002
Use of antihypertensive drugs	3.78 (1.76–8.08)	.001
Use of RAS blockers	0.37 (0.18–0.73)	.005
Use of lipid-lowering drugs	1.43 (0.76–2.67)	.27

Abbreviations: HbA_1c_, glycated hemoglobin; RAS blockers, renin-angiotensin system blockers, including angiotensin converting enzyme inhibitors and angiotensin receptor antagonists.

a Calculated as weight in kilograms divided by height in meters squared.

Multivariate logistic analysis was conducted to adjust for confounding factors. We found that patients with early-onset type 2 diabetes had 2.3-fold (95% CI, 1.2–4.4) higher risk of ESRD compared with patients with late-onset type 2 diabetes after adjustment for sex (our model 1), and the difference was significant (Table 3). Further adjustment for other metabolic factors (BMI, SBP, DBP, TC, TG, LDL-C, HDL-C, HbA_1c_, smoking history, drinking history, and hypertension history) in patients with early-onset type 2 diabetes was associated with 4.5-fold (95% CI, 2.0–10.0) increased risk for ESRD compared with patients with late-onset type 2 diabetes (our model 2) (Table 3). The increased risk of ESRD was only slightly attenuated after further adjustment for drug factors (oral hypoglycemic drugs, insulin therapy, antihypertensive drugs, RAS blockers, lipid-lowering drugs) (our model 3) (OR, 4.5; 95% CI, 1.9–10.6) (Table 3). After further adjustment for duration of diabetes, the risk of ESRD decreased somewhat, but the overall correlation remained significant (our model 4) (OR, 3.6; 95% CI, 1.5–8.7) (Table 3).

**Table 3:** Odds Ratio of Early-Onset Type 2 Diabetes for End Stage Renal Disease in Patients With Diabetic Kidney Disease, The First Affiliated Hospital of Chongqing Medical University, China, 2014–2018

Model	Odds Ratio (95% Confidence Interval)	*P* value
Model 1, early vs late onset^a^	2.3 (1.2–4.4)	.011
Model 2, early vs late onset^b^	4.5 (2.0–10.0)	<.001
Model 3, early vs late onset^c^	4.5 (1.9–10.6)	.001
Model 4, early vs late onset^d^	3.6 (1.5–8.7)	.005

a Adjusted for sex.

b Further adjusted for traditional metabolic factors, including body mass index, systolic blood pressure, diastolic blood pressure, total cholesterol, total triglyceride, high density lipoprotein cholesterol, low density lipoprotein cholesterol, glycated hemoglobin, smoking history, drinking history, and hypertension history in addition to sex.

c Further adjusted for use of oral hypoglycemic drugs, insulin, antihypertensive drugs, renin-angiotensin system blockers (including angiotensin converting enzyme inhibitors and angiotensin receptor antagonists), and lipid-lowering drugs in addition to the variables in model 2.

d Further adjusted for the duration of diabetes in addition to the variables in model 3.

## Discussion

In our study, we demonstrated for the first time that early-onset diabetes is associated with the risk of ESRD in the patients with DKD. This finding remains significant after adjusting for potential confounding factors such as duration of diabetes, sex, traditional metabolic factors, and drug factors, suggesting that early-onset diabetes is an independent risk factor for ESRD in patients with DKD.

The onset age of type 2 diabetes has been decreasing, and the prevalence of early-onset type 2 diabetes is increasing rapidly in both developed and developing countries (21,22). The International Diabetes Federation estimated that roughly 63 million young adults aged 20 to 39 years had type 2 diabetes worldwide in 2013; early-onset diabetes accounted for 16% of total adults with type 2 diabetes (23). In our study, early-onset type 2 diabetes accounted for 13.7% of all recruited patients. With the increased prevalence of early-onset diabetes, the incidence of diabetic complication such as cardiovascular and cerebrovascular diseases, chronic kidney disease, and even ESRD is gradually increasing (7–10). A longitudinal population-based study in the United States reported that onset of type 2 diabetes in participants younger than age 20 years was associated with a nearly fivefold increase in the incidence of ESRD between 25 and 54 years of age compared with a later onset of type 2 diabetes (7). Data from a prospective cohort study in Singapore also showed that early-onset type 2 diabetes was associated with a substantially higher risk of progressive chronic kidney disease compared with late-onset type 2 diabetes (24). These studies agree with our data showing that early-onset type 2 diabetes patients have increased risk of ESRD. However, a cross-sectional study of the Joint Asia Diabetes Evaluation cohort study found that although there was a higher proportion of ESRD in the early-onset diabetes group, this difference was not significant (12). In addition, in the United States study (7), Pavkov et al also said that the effect of youth-onset type 2 diabetes increasing the risk of ESRD will disappear after adjusting for age, sex, diabetes duration, and other factors. Pavkov and colleagues conducted their research in the Pima Indian population, which might be quite different from our hospital-based study. Also, Pavkov and colleagues limited the analysis of ESRD to young and middle-aged Pima Indians, because data were insufficient to compute adjusted incidence rates in the youth-onset group older than age 55 years and in the older-onset group younger than age 25 years. This lack of data might introduce selection bias. Besides, our research object was patients with DKD and the population spectrum is narrower, which is also a possible reason for the inconsistent results.

The mechanisms by which early-onset diabetes increases the risk of DKD progression have not been fully clear. Several epidemiologic studies have found that the increased risk of ESRD associated with early-onset type 2 diabetes can be attributed to the prolonged duration of diabetes and subsequent deterioration of metabolic status. For example, a cross-sectional survey in 77 tertiary hospitals in China found that early-onset diabetes greatly increased the prevalence of microvascular diseases compared with late-onset diabetes after adjusting for age, sex, and traditional risk factors, but the difference was not significant after adjusting for disease duration (9). Prolonged diabetes duration might lead to long-term exposure to hyperglycemic conditions, which might lead to reduced β-cell function, increased oxidative stress, and activation of the RAS (25), and then promote the progression of diabetes complications and increase the risk of ESRD. However, our study found that early-onset diabetes is an independent risk factor for ESRD, even after adjusting for duration of diabetes. This may be related to genetic variation and strong family history of diabetes. Kong et al found that early-onset diabetes had a strong genetic predisposition, which could be mainly linked to β-cell function (26). Other investigations showed that HNF4α protein expression was repressed and T allele rs3760106 variation of PRKCB1 and the G allele rs2575390 variation were correlated with the occurrence of DKD in patients with early-onset diabetes (27,28). Protein kinase C-β encoded by PRKCB1 gene is closely related to the occurrence of macroalbuminuria, eGFR decline, and ESRD (29,30). The above evidence suggests that diabetes duration and genetic susceptibility play an important role in the occurrence of early-onset diabetes and the development of vascular complications. Other known mechanisms include socioeconomic and psychological-behavioral. Whether other mechanisms exist needs further investigation.

Our study has the following strengths. First, the relationship between early-onset type 2 diabetes and ESRD has been reported for the first time in the DKD population. With the increased prevalence of early-onset diabetes, our research has the important clinical implication that we need to pay more attention to the subset of DKD patients with early-onset diabetes, and apply more stringent metabolic targets to delay DKD progression. Second, we studied a large sample. Third, our study minimizes the effect of duration or other risk factors by using statistical adjustments.

Some limitations of the study are worth mentioning. First, our study was cross-sectional. Prospective cohort studies to explore the cause–effect association between early-onset type 2 diabetes and ESRD in patients with DKD are needed. Second, patients were selected among hospitalized patients in a single center, which might lead to a selection bias. Also, the results need to be verified by a larger sample size from a multicenter population. In addition, we cannot know the precise onset age of type 2 diabetes because of the insidious onset of this disease. The observed onset age may be older than the real onset age.

Our study provides information on the effect of the age of diagnosis of diabetes on the development of ESRD in patients with DKD. Compared with type 2 diabetes of typical onset in middle-aged and elderly patients, patients with early-onset diabetes have poorer blood lipids and blood glucose status and a higher prevalence of proteinuria and ESRD. These perspectives highlight the growing imperative to direct attention toward early-onset type 2 diabetes and for effective interventions to be applied before middle age.
